# A retrospective review of the contribution of rare diseases to paediatric mortality in Ireland

**DOI:** 10.1186/s13023-020-01574-7

**Published:** 2020-11-04

**Authors:** Emer Gunne, Cliona McGarvey, Karina Hamilton, Eileen Treacy, Deborah M. Lambert, Sally Ann Lynch

**Affiliations:** 1 Children’s Health Ireland, Temple Street, Dublin, Republic of Ireland; 2 National Paediatric Mortality Register, Dublin, Republic of Ireland; 3 National Rare Disease Office, Mater Misericordiae University Hospital, Dublin, Republic of Ireland

**Keywords:** Rare disease, Paediatric, Mortality, Health care burden, Epidemiology

## Abstract

**Aims:**

To ascertain the number of paediatric deaths (0–14 years) with an underlying rare disease in the Republic of Ireland between the years 2006–2016, and to analyse bed usage by a paediatric cohort of rare disease inpatients prior to in-hospital death.

**Background:**

Rare diseases are often chronically debilitating and sometimes life-threatening diseases, with the majority (69.9%) of rare diseases being of paediatric onset. The Orphanet database contains information on 6172 unique rare diseases. Under-representation of rare diseases in hospital healthcare coding systems leads to a paucity of rare disease epidemiological data required for healthcare planning. Studies have cited variable incidence rates for rare disease, however the burden of rare diseases to healthcare services still remains unclear. This study represents a thorough effort to identify the percentage of child mortality and paediatric bed usage attributable to rare diseases in the Republic of Ireland, thus addressing a major gap in the rare disease field.

**Methods:**

Retrospective analysis of paediatric death registration details for the Republic of Ireland in the 11-year period 2006–2016 from the National Paediatric Mortality Register. Data was subcategorised as Neonatal (0–28 days), Post Neonatal (29 days < 1 year) and older (1–14 years). Bed usage data (ICD-10 code, narrative and usage) of paediatric inpatients who died during hospitalisation from January 2015 to December 2016 was extracted from the National Quality Assurance Improvement System of in-patient data. Orphacodes were assigned to rare disease cases from ICD-10 codes or diagnostic narrative of both datasets.

**Results:**

There were 4044 deaths registered from 2006–2016, aged < 15 years, of these 2368 (58.6%) had an underlying rare disease. Stratifying by age group; 55.6% (1140/2050) of neonatal deaths had a rare disease, 57.8% (450/778) post-neonatal, and 64% (778/1216) of children aged 1–14 years. Mortality coding using ICD-10 codes identified 42% of rare disease cases with the remainder identified using death certificate narrative records. Rare disease patients occupied 87% of bed days used by children < 15 years who died during hospitalisation from January 2015 to December 2016.

**Conclusion:**

Additional routine rare disease coding is necessary to identify rare diseases within Irish healthcare systems to enable better healthcare planning. Rare disease patients are overrepresented in paediatric mortality statistics and in-patient length of stay during hospital admission prior to death.

## Background

The Orphanet database contains information on 6172 unique rare diseases (RDs) [1, 2]. The majority of these often chronic and sometimes life-limiting RDs (69.9%) are of paediatric onset [2].

The European Commission’s report on RDs [3], published in 2008, recommended the need for a coherent strategy to develop cooperation, coordination and regulation for RDs at European Union level. Subsequently, the Council of Ministers of the European Union called for countries to adopt a national plan or strategy aimed at guiding and structuring actions in the field of RDs within the framework of their health and social systems (2009) [4]. The UK Strategy for RDs (2013) [5] and The Irish National Plan for RDs [6] (2014–2018), both recommended the undertaking of epidemiological research studies to identify patients and burden of disease, highlighting the requirement for the implementation of RD coding. Currently there is no system to quantify the number of people affected by RDs and little is known about the RD diagnosis, age profile, mortality or morbidity in the Republic of Ireland (ROI).

During the period 2006–2016, a total of 774,048 births were registered in the ROI [7]. Paediatric care in the Irish healthcare system extends until the 16th birthday, after which care is obtained in the adult healthcare system. It is widely recognized that mortality patterns change after the age of 15 years [8]. The Census 2011 and Census 2016 showed n = 979,590 and n = 1,006,552 children aged 14 and under respectively, with an infant mortality rate of 3.0 per 1000 live births [7] recorded in 2016.

Two national datasets can be used to examine paediatric mortality in the ROI. Paediatric mortality data is collected by the Irish National Paediatric Mortality Registry (INPMR) [9] from data provided from the Central Statistics Office (CSO) on a quarterly basis in the form of encrypted microdata files. Registration details of all deaths in Ireland are forwarded from the General Registration Office to the CSO, which collects and analyses vital statistics on behalf of the Minister for Health. Principle variables included in the CSO mortality database are age, gender, place and cause of death. Each registered death is attributed an underlying cause of death code according to the rules and guidelines published by the World Health Organisation in the International Classification of Diseases ICD-10 classification. The US Centre of Disease Control-developed Medical Mortality Data System (MMDS) suite of software was used by the CSO for the automated coding of deaths in the ROI until 2018. Data for in-patient hospital use in the ROI is collected by the National Quality Assurance & Improvement System (NQAIS) Clinical [10]. NQAIS Clinical is a Health Atlas Ireland online reporting system that analyses ROI Hospital-In-Patient Enquiry (HIPE) data. HIPE records are created following patient discharge from hospital, recording diagnostic, procedural and administrative details relating to their care during an admission.

It has been recognized for over 10 years that the majority of RDs are not captured in ICD-10 codes with the result that the health burden of RDs is invisible in health information systems [11].

The Orphacodes RD nomenclature, curated by Orphanet, is designed to capture all RD diagnoses [11, 12]. ICD-10 has only 500 RDs represented, with only 250 with an ICD10 code mapping exactly to one RD by a specific code [11]. Through the work of the Topic of Advisory Group for Rare Diseases, the ICD-11 contains representation of 5400 RDs, ten-fold more than the ICD-10. However, it is understood that it will not be possible for the ICD-11 to continue to evolve to incorporate further RD definitions in this rapidly evolving field [12], and Orphacoding, which is updated on an ongoing basis to keep apace of medical developments, is still considered to be the gold standard.

RD patients’ care is more efficient if managed in centralised centres of expertise [3]. These centres linked by European Reference Networks (ERNs) endeavor to facilitate equal access to accurate information, appropriate and timely diagnosis and high quality of care for RD patients [3]. In addition, health funding systems, such as Activity Based Funding introduced in January 2016 in the ROI, are insufficient for budgeting/costing highly specialised care. Costs associated with the provision of treatment in specialist paediatric hospitals are funded through a co-payment mechanism and are not included in the Activity Based Funding Diagnosis Related Group admitted patient price list [13]. By identifying RD cases within hospital coding systems, it may be possible to target specific funding to improve care for this cohort.

The objective of this study is to ascertain the number of paediatric deaths (0–14 years) with an underlying RD in the ROI between the years 2006–2016, and to analyse bed usage by a paediatric cohort of RD inpatients prior to in-hospital death.

### Methods

Details of all deaths in children, registered in the ROI in the 11-year period from January 2006 to December 2016 were obtained by the Irish National Paediatric Mortality Registry (INPMR) [9] at Temple Street Children’s University Hospital – Children’s Health Ireland. The details on all deaths registered in the ROI during the 11-year period 2006–2016 were reviewed and classified by age of death. To aid with international comparison, cases were grouped into Neonatal (0–28 days), Post-Neonatal (29 days < 1 year), children aged 1–4 years, 5–9 years and 10–14 years. Review of ICD-10 codes and narrative descriptions from death registration was undertaken to identify RD cases. RD cases were assigned an Orphacode at the specific disorder level where possible; otherwise, an Orphacode for the broader hierarchical disorder group was used. To provide national mortality context, the Crude Mortality Rate (per 100,000 births) was calculated and stratified by age group for 2006 and 2016 from the CSO census and mortality data [7], included in Table 1.

**Table 1:** Classification of all registered deaths in children < 15 years in Ireland for the period 2006–2016 by age group and within age groups those identified as having a rare disease

Age group	Total number of cases n (%)	Rare disease cases n (%)	Rare disease cases as a % of total n (%)	Crude mortality rate per 100,000 [7]	
	2006–2016	2006–2016	2006–2016	2006	2016
Neonatal	2050 (51%)	1140 (41%)	1140/2050 (55.6%)	–	–
Post-neonatal	778 (19%)	450 (19%)	450/778 (57.8%)	–	–
Age 0–1 years	2828 (70%)	1590 (60%)	1590/2828 (55.2%)	389.8	311.9
Age 1–4 years	527 (13%)	337 (14.2%)	337/527 (63.9%)	15.8	11.5
Age 5–9 years	316 (8%)	219 (9.2%)	219/316 (69.3%)	11.1	3.7
Age 10–14 years	373 (9%)	222 (9.3%)	222/373 (59.5%)	12.8	9.4
Total	4044 (100%)	2368 (100%)	2368/4044 (58.6%)*	41.1	26.7

^*^p < 0.005 for the Z score testing the hypothesis that there is a difference in the proportion of children with a RD in the mortality register compared to the expected proportion from children with RD in the general population

Length of stay of paediatric patients who died during hospitalisation, was assessed by analysing data from the National Quality Assurance & Improvement System (NQAIS) Clinical [10]. From NQAIS Clinical a report of all children less than 15 years old who were discharged deceased between 1st of January 2015 and 31st of December 2016 in the ROI was reviewed. ICD-10 codes and diagnostic narrative was analysed to ascertain RD cases. Length of stay, including intensive care unit usage, was calculated.

The expected proportion of children with an RD was derived from Orphanet data calculated in Nguengang-Wakap et al. [2], using the figures of 5.9% for the population prevalence of RD with 69.9% of RDs having a childhood onset giving an estimated 4.1% of the paediatric population prevalence of RDs. Z score statistics were used to test the hypotheses of the proportion of children with a RD observed compared to the expected proportion of children with RDs in the datasets analysed.

### Results

Data from INMPR (Table 1) shows that of all deaths (n = 4044) aged 0–14 years registered in the ROI during the period, (2006–2016), 58.6% (n = 2368) had a RD diagnosis. When stratified by age category: neonates, 55.6% (1140/2050), post-neonates, 57.8% (450/778), children aged 1–4 years, 63.9% (337/527), children aged 5–9 years, 69.3% (219/316), children aged 10–14 years, 59.5% (222/373) had a RD. There was a significantly higher proportion of RD patients among registered paediatric deaths (58.6%) than expected (4.1%) Z = 52.7876, *p* < 0.00001. There was no analysis of significance undertaken by stratified age groups as there are no estimates of RD onset in each of these age categories (Table 2). The most common RD diagnoses across all age groups include trisomy 18 (n = 147, 6.2%), hypoplastic left heart (n = 96, 4.1%), anencephaly (n = 94, 3.9%) and trisomy 13 (n = 77, 3.2%). Deaths due to birth defects (n = 588, 51.5%) and rare infections (n = 116, 10.1%) were the most common causes among neonates. Of the birth defect cases, 73/588 (12.4%) had multiple congenital anomalies. Of the infectious cases, the highest number were due to sepsis in premature infants (n = 102, 87.9%). Rare genetic disorders had the highest category in the post-neonatal period and rare neoplastic disorders were most frequently represented in the older age group of 1–14 years. Within the category ‘Other’, there were 54 (2.2%) cases of cerebral palsy, 57 (2.4%) cases of cardiomyopathy and 27 (1.1%) cases of epilepsy.

**Table 2:** Categorisation of all registered deaths < 15 years by Rare Disease category and age 2006–2016

	Neonatal (0–28 days)	Post-neonatal (29 days < 1 year)	Children (1–14 years)	Total
CATEGORY	Number of cases (%)	Number of cases (%)	Number of cases (%)	
Rare disease	(n)	(n)	(n)	
**BIRTH DEFECTS**	**588 [51.58%]**	**147 [32.67%]**	**93 [11.95%]**	**828**
**Rare development defect during embryogenesis**				
Anencephaly	(93)	(< 5)	–	
Multiple congenital anomalies	(73)	(11)	–	
Renal agenesis	(52)	–	–	
Congenital diaphragmatic hernia	(38)	(10)	(< 5)	
Hydrops fetalis	(13)	–	–	
Holoprosencephaly	(11)	(< 5)	(< 5)	
Omphalocele	(9)	–	–	
VACTERL/VATER association	(8)	–	(< 5)	
Esophageal atresia	(8)	(< 5)	–	
Spina bifida	(8)	(< 5)	(7)	
Congenital hydrocephalus	(6)	(< 5)	(5)	
Renal hypoplasia	(5)	–	(< 5)	
Other defect during embryogenesis	(116)	(28)	(4)	
**Rare congenital heart malformations**				
Hypoplastic left heart	(67)	(22)	(7)	
Tetralogy of fallot	(6)	(< 5)	(< 5)	
Truncus arteriosus	(5)	(< 5)	–	
Ebstein malformation	(< 5)	(< 5)	(< 5)	
Congenital valvular dysplasia	(< 5)	(< 5)	–	
Aorta coarctation	(< 5)	(< 5)	(< 5)	
Other rare congenital heart malformation	(61)	(53)	(19)	
**RARE GENETIC**	**346 [30.3%]**	**192** **[42.67%]**	**249 [32.01%]**	**787**
**Chromosomal anomaly**				
Trisomy 18	(114)	(31)	(< 5)	
Trisomy 21	(29)	(36)	(22)	
Trisomy 13	(64)	(10)	(< 5)	
22q11.2 deletion syndrome	(6)	(< 5)	(< 5)	
Pallister-Killian syndrome	(< 5)	(< 5)	(< 5)	
Triploidy	(< 5)	–	–	
Wolf-Hirschhorn syndrome	(< 5)	–	(< 5)	
Turner syndrome	(< 5)	(< 5)	–	
Other chromosomal anomaly	(21)	(16)	(27)	
**Inborn errors of metabolism**				
Mitochondrial disease	(6)	(7)	(21)	
Alpers syndrome	–	–	(11)	
Leigh syndrome	(< 5)	(< 5)	(10)	
Batten disease	–	–	(< 5)	
Hurler syndrome	–	(< 5)	(< 5)	
Other inborn errors of metabolism	(9)	(24)	(30)	
**Rare neurologic disease**				
Schizencephaly	–	–	(6)	
Spinal Muscular Atrophy	–	(17)	(6)	
Muscular dystrophy	(< 5)	–	(6)	
Rett syndrome	–	–	(6)	
Aicardi-Goutieres Syndrome	–	–	(< 5)	
Other rare neurologic disease	–	(16)	(29)	
Cystic Fibrosis	–	(<5)	(11)	
Rare endocrine disease	(< 5)	–	(5)	
Rare hematologic disease	(5)	(< 5)	(5)	
Rare immune disease	–	(5)		
Other rare genetic	(86)	(24)	(29)	
**RARE NEOPLASTIC**	**10 [0.88%]**	**11 [2.44%]**	**265 [34.06%]**	**286**
Acute lymphoblastic leukemia	–	(< 5)	(27)	
Neuroblastoma	–	–	(26)	
Rhabdoid tumour	–	(< 5)	(6)	
Medulloblastoma	(< 5)	–	(12)	
Glioma	(< 5)	(< 5)	(11)	
Astrocytoma	(< 5)	(< 5)	(12)	
Teratoma	(< 5)	–	–	
Other rare neoplastic	(4)	(2)	(171)	
**RARE INFECTIOUS**	**116 [10.18%]**	**43 [9.56%]**	**39 [5.01%]**	**198**
Sepsis in premature infants	(102)	(27)	(< 5)	
Meningitis	(< 5)	(9)	28	
Pertussis	–	(6)	–	
Congenital Herpes simplex virus infection	(< 5)	–	(< 5)	
Congenital toxoplasmosis	(< 5)	–	–	
Fetal cytomegalovirus syndrome	(< 5)	–	(< 5)	
Other rare infectious	(4)	(1)	(8)	
**OTHER**	**80 [7.02%]**	**57 [12.67%]**	**132 [16.97%]**	**269**
Cardiomyopathy	(12)	(20)	(25)	
Cerebral Palsy	–	(< 5)	(52)	
Epilepsy	–	(< 5)	(23)	
Other rare disease	(68)	(30)	(32)	
**TOTAL**	**1140 [100%]**	**450 [100%]**	**778 [100%]**	**2368**

Categories where case numbers are less than 5 have been accounted for as < 5 to avoid disclosure issues

A comparison was undertaken of the RD cases identified in this study (n = 2368) versus the number of RD cases identified if only the ICD-10 coding was used. Use of the ICD-10 code in isolation of the narrative record identified only 995 rare disease cases (42%), meaning that Orphacodes use would have been required to capture 1373 (58%) of RD cases.

NQAIS Clinical summary analysis of the in-patient healthcare burden of RD patients is shown in Table 3. RD patients accounted for 66% of the children less than 15 years who died in hospital during the 2-year study period, significantly higher than the expected 4.1% (Z = 46.6 *p* < 0.0001). These RD patients had significantly longer length of stay than the non-RD in-hospital deaths. The median length of stay for RD patients was 5 days while it was 2 days for non-RD cases. The 66% of the patients who had a RD, used 87% of all bed days (Z = 7.96 *p* < 0.0001) and 83% of the intensive care unit days (Z = 5.17 *p* < 0.0001) in the period of hospitalisation prior to their death.

**Table 3:** National hospital data of children < 15 years discharged deceased in Ireland for the period January 2015-December 2016 with analysis of bed usage

	Number of deaths number of patients (%)	Total length of stay (LOS) number of days (%) [Median LOS]	Standard bed LOS number of days (%)	ICU total LOS number of days (%)
All patients	365 (100%)	5566.5 (100%) [3 days]	1527.5 (100%)	4039 (100%)
RD patients	240 (66%)	4845.0 (87%) [5 days]	1504.0 (98.5%)	3341 (83%)
Non-RD patients	125 (34%)	721.5 (13%) [2 days]	23.5 (1.5%)	698 (17%)
Z score testing	Z = 46.6 *p* < 0.0001*	Z = 7.96 *p* < 0.0001*	Z = 52.17 *p* < 0.00001*	Z = 5.17 *p* < 0.0001*

^*^p < 0.0001 for the Z score testing the hypothesis that there is a difference in the proportion of children with a RD in national hospital data compared to the expected proportion from children with RD in the general population

An estimate of the completeness of the bed usage data in this study can be derived by comparing the deaths per year in the data sources. National Mortality data (4044 cases/11 years) gives a mean of 368 deaths per year compared to the NQAIS bed usage data (365 cases/2 years) giving 182 deaths per year; suggesting that NQAIS data captures approximately 49% of the bed usage data related to paediatric deaths between the years 2015–2016.

### Discussion

RD epidemiological studies, in the ROI, are time-consuming given that; (a) there is no centralised registry of RDs in the ROI and (b) the coding and other data sources require review of narrative records. This leads to poor recognition of RDs in the Irish healthcare system. The limited capacity to trace RDs within mainstream Healthcare Information Systems is due to the use of the ICD-10 coding system, which does not sufficiently represent RDs. This study has demonstrated that only 42% of RD cases are detectable using ICD10 coding. The main deficiency, from a RD epidemiology perspective, is when processing a death certificate because the mortality software looks at all the diagnostic expressions on a death certificate and then selects the underlying cause of death according to the guidelines set out in the ICD-10 classification. For example, a case with narrative indicating the patient had both a rare Mitochondrial disease and influenza was coded ICD-10 code J11.00, (Influenza), as the cause of death, therefore the RD diagnosis was not represented in CSO analysis.

We used the Orphacode nomenclature for consistency, as it is the only nomenclature to capture all individual RDs [11]. To allow for international comparison, all diseases classed as RD in Europe (prevalence of less than 1 per 2,000) were included in the study despite having a known prevalence of greater than 1 per 2,000 in Ireland, such as cystic fibrosis, Down syndrome and neural tube defects. Use of the ORPHAcode system also gave rise to coding challenges with some ambiguous coding, which will be addressed by the new Orphacoding helpdesk [14]. The addition of an Orphacode to the nascent ROI Electronic Health Records would allow better identification and costing of these cases, and has been endorsed in the 2019 Model of Care for Rare Diseases [15].

We recognize that other countries undertaking a similar study may find a lower prevalence of paediatric RDs. EUROCAT data clearly demonstrates that termination of pregnancy for congenital anomalies in the rest of Europe was 10 times the Irish rate in 2011 and 4.3 times in 2016 [16]. Second trimester fetal anomaly scans are not routine practice in the ROI and termination of pregnancy was against the constitution during the study period, until January 2019 [17]. Therefore, in neighbouring European countries the contribution of the more common birth defects which can be detected prenatally leading to post-natal and paediatric mortality would be expected to be significantly less than in the ROI.

It is clear that a significant proportion of RDs have a genetic basis, with the exception of rare infectious causes. In addition to the known genetic basis of many of the congenital anomalies [18] and rare tumours; within the category ‘other’, there were cases of cerebral palsy [19], cardiomyopathy [20] and epilepsy [17] which are known to have a significant genetic contribution. Ongoing improvements and availability of next generation sequencing technology continues to reveal a genetic component of many of these cases. Therefore, the contribution of genetic disorders to RDs is likely to continue to rise for the foreseeable future [21].

Our review demonstrates that RD cases are over-represented among ROI paediatric mortality data. Whilst the breadth of disorders is vast and any investment in managing these disorders will need largely to be tailored to the specific symptomatology of each patient group, these are all complex, often chronic disorders that require highly specialised care. Quantifying their number and healthcare burden will help inform those working in public health. Health care plans for RDs requires a coordinated structure in which to operate and this is better informed by good epidemiological data [22].

### Study limitations

No trend over time analysis was possible due to the small numbers of RD cases detected by age group, by year. During the study period, the percentage of the population in the 0–14 age group remained steady (20.4% in 2006, 21.3% in 2011 and 21.1% in 2016) [7] although in 2008–2012 there was an increase in birth rate that influenced the age distribution within the 0–14 age group from 2008–2016 [7]. The mortality rate of paediatric RDs cannot be calculated as there is no systemic collection of RD diagnoses in Ireland.

There are limitations to the in-patient data used for the length of stay analysis. However, another study [23] which looked at the health system burden of RD had similar findings, showing that length of stay is higher in both paediatric and neonatal genetic disease compared to non-genetic disease. In our study data for bed usage analysis only captured RD patients who passed away in hospital using the search criterion ‘discharged dead’, which accounted for an estimated 49% of all deaths in this study period. We recognise that patients with life-limiting disorders may have died at home or in palliative care services and will therefore not have been captured using our methodology representing an under-ascertainment. While there is no national data on paediatric deaths outside of hospital, provisional incomplete data capture suggests that there are at least 27 paediatric palliative care deaths in home or in hospital per year in the ROI (F. McElligott, personal communication).

The pattern of length of stay for RD patients will be in constant flux, reflecting demographic changes and our study captures only a snapshot of the issue. The emergence of new RD treatments such as nusinersen for Spinal muscular atrophy may result in increased length of in hospital stay. In contrast, the availability of termination of pregnancy in the ROI (January 2019) [17] and the increasing availability of prenatal versus postnatal diagnosis is likely to lead to a reduction in bed usage from lethal congenital anomalies.

### Conclusions

Public policies to improve access to services for people with RDs require local epidemiological data in order to inform planning. It is evident from this study (of official death registration information) that a large portion of paediatric mortality cases (58.6%) are due to an underlying RD. Addition of Orphacodes to eHealth records would allow RD cases to be reported and costed easily. The most common causes of RD mortality include Chromosomal anomaly (16.8%), Neoplastic (12.0%), congenital heart malformations (11.0%), and Cardiomyopathies (2.4%). In the 2-year study period, RD patients used the majority (87%) of bed days of all children < 15 years in the time period leading up to their death in hospital.

## Data Availability

All grouped data generated or analysed during this study are included in this published article. No individual data is available to protect the recognition of individual patients.

## Abbreviations

CSO: Central Statistics Office
EUROCAT: European network of population-based registries for the epidemiological surveillance of congenital anomalies
ICD-10: International Statistical Classification of Diseases and Related Health Problems nomenclature, 10th version
HIPE: Hospital-In-Patient Enquiry
INPMR: Irish national paediatric mortality registry
Iris: Automated, interactive mortality coding system
LOS: Length of stay
MMDS: US Centre of Disease Control-developed Medical Mortality Data System
NQAIS: National Quality Assurance & Improvement System
RD: Rare disease
ROI: Republic of Ireland
